# Respiratory-Induced Amplitude Modulation of Forcecardiography Signals

**DOI:** 10.3390/bioengineering9090444

**Published:** 2022-09-07

**Authors:** Jessica Centracchio, Emilio Andreozzi, Daniele Esposito, Gaetano D. Gargiulo

**Affiliations:** 1 Department of Electrical Engineering and Information Technologies, University of Naples Federico II, Via Claudio, 80125 Napoli, Italy; 2 School of Engineering, Design and Built Environment, Western Sydney University, Penrith, NSW 2751, Australia

**Keywords:** forcecardiography, respiration, cardiac monitoring, cardiomechanical signals, mechanocardiography

## Abstract

Forcecardiography (FCG) is a novel technique that records the weak forces induced on the chest wall by cardio-respiratory activity, by using specific force sensors. FCG sensors feature a wide frequency band, which allows us to capture respiration, heart wall motion, heart valves opening and closing (similar to the Seismocardiogram, SCG) and heart sounds, all simultaneously from a single contact point on the chest. As a result, the raw FCG sensors signals exhibit a large component related to the respiratory activity, referred to as a Forcerespirogram (FRG), with a much smaller, superimposed component related to the cardiac activity (the actual FCG) that contains both infrasonic vibrations, referred to as LF-FCG and HF-FCG, and heart sounds. Although respiration can be readily monitored by extracting the very low-frequency component of the raw FCG signal (FRG), it has been observed that the respiratory activity also influences other FCG components, particularly causing amplitude modulations (AM). This preliminary study aimed to assess the consistency of the amplitude modulations of the LF-FCG and HF-FCG signals within the respiratory cycle. A retrospective analysis was performed on the FCG signals acquired in a previous study on six healthy subjects at rest, during quiet breathing. To this aim, the AM of LF-FCG and HF-FCG were first extracted via a linear envelope (LE) operation, consisting of rectification followed by low-pass filtering; then, the inspiratory peaks were located both in the LE of LF-FCG and HF-FCG, and in the reference respiratory signal (FRG). Finally, the inter-breath intervals were extracted from the obtained inspiratory peaks, and further analyzed via statistical analyses. The AM of HF-FCG exhibited higher consistency within the respiratory cycle, as compared to the LF-FCG. Indeed, the inspiratory peaks were recognized with a sensitivity and positive predictive value (PPV) in excess of 99% in the LE of HF-FCG, and with a sensitivity and PPV of 96.7% and 92.6%, respectively, in the LE of LF-FCG. In addition, the inter-breath intervals estimated from the HF-FCG scored a higher R^2^ value (0.95 vs. 0.86) and lower limits of agreement (± 0.710 s vs. ±1.34 s) as compared to LF-FCG, by considering those extracted from the FRG as the reference. The obtained results are consistent with those observed in previous studies on SCG. A possible explanation of these results was discussed. However, the preliminary results obtained in this study must be confirmed on a larger cohort of subjects and in different experimental conditions.

## 1. Introduction

The heart accomplishes its function of pumping blood through the cardiovascular system via the contraction of the atria and ventricles. The mechanical contraction is accompanied by the gross motion of the heart, as a result of its morphological changes. Specifically, due to the recoil effect that causes the stretch of elastic tissues, the heart contracts and propels itself towards the apex (i.e., downward) during systole, whereas it relaxes and springs back towards the base (i.e., upward) during diastole [1]. However, heart motion also depends on the mechanical coupling to adjacent structures [2], such as the lungs; hence, it is affected by respiration. Respiration is characterized by the following two motion components: thoracic, due to the movement of the rib cage, and abdominal, due to the displacement of the diaphragm [3]. Indeed, during inspiration, the rib cage expands, while the diaphragm moves downward. On the contrary, during expiration, the rib cage releases, while the diaphragm moves upward. The heart sits on the diaphragm and is in contact with the lungs, so it moves with them during breathing acts. It is well known from the literature that the heart movement during respiration essentially occurs along the superior–inferior direction, is linearly related to the displacement of the diaphragm, and can be considered approximately as a global longitudinal translation [4].

The mechanical activity of the heart and adjacent structures generates vibrations that propagate to the surrounding tissues, eventually reaching the surface, where they induce small movements of the chest wall.

Since the second half of the 19th century, several non-invasive techniques have been introduced to monitor these tiny movements, by leveraging different physical principles. Apexcardiography (ACG) [5,6], Seismocardiography (SCG) [7,8,9,10,11], and Phonocardiography (PCG) [12,13] have been the most successful, among the techniques proposed at the outset. ACG and SCG both record the infrasonic components of precordial vibrations, while PCG captures the sonic components, commonly known as heart sounds. ACG and SCG lost their appeal, because of the cumbersome instrumentation required, and the outbreak of ultrasound imaging, which brought outstanding diagnostic capabilities by allowing physicians to actually look inside the human body. Recent advancements in microelectromechanical systems (MEMS) have rejuvenated the research on SCG, which is now ranked among the most wearable monitoring technologies and is performed via small inertial measurement units (IMU). Since IMUs usually also include gyroscopes, new techniques have recently been proposed, namely Gyrocardiography (GCG), which captures the rotational components of local cardiac vibrations [14,15]. The combined use of SCG and GCG, also from multiple sites, has further been investigated [16,17,18]. SCG and GCG have proven useful as wearable surrogates for echocardiography in estimating cardiac time intervals, which provide clinically relevant information for diagnosis and management of various cardiac pathologies [7,8,9,10,11,19].

Forcecardiography (FCG) has been proposed as a novel, non-invasive technique to measure the local vibrations of the chest wall via specific force sensors [20,21,22], which have also been used to monitor muscle contraction [23,24,25]. FCG sensors record vibrations over a wide frequency range, which provide information on both cardiac and respiratory activity from a single contact point on the chest. The raw FCG sensor signal essentially consists of a very low-frequency component related to respiration, referred to as the Forcerespirogram (FRG), and a further component that reflects the cardiac activity, which is the actual Forcecardiogram [21,22]. The latter features infrasonic cardiac vibrations that can be further divided into low-frequency (LF-FCG) and high-frequency (HF-FCG) components. LF-FCG seems to be related to ventricular emptying and filling events, thus potentially carrying information on stroke volume variations [20,21,22]. Moreover, a specific numerical procedure based on double integration was devised to extract the same information from the SCG signal. This procedure yields a new displacement signal (DSCG) that features a low-frequency component (LF-DSCG) very similar to LF-FCG [26]. HF-FCG captures SCG-like information. In fact, it shares a very high similarity with SCG [20,22], and provides timings of aortic valve opening events and estimates of pre-ejection periods (PEPs) with high accuracy and precision, as compared to SCG [27]. In addition, piezoelectric FCG sensors have also shown their ability to capture heart sounds, which are contained in the sonic component of the FCG signal, referred to as HS-FCG [22].

The sonic and infrasonic components of the FCG signal reflect mechanical waves that propagate from the heart to the chest surface, where they are sensed by FCG sensors placed in fixed sites of the chest wall. Since the heart experiences upward and downward motions during respiration, these mechanical waves turn out to be generated by a moving source and propagate along paths of varying length and physical properties. Depending on the position of the measurement site over the chest, the varying distance from the source is likely to cause a likewise variable attenuation of the amplitude of the mechanical waves, thus resulting in amplitude modulation of the FCG signal components.

To date, this effect of respiration on FCG signals has not been investigated yet. This preliminary study addresses this issue, by focusing on the respiratory-related amplitude modulation of LF-FCG and HF-FCG signals sensed from a specific site over the chest.

## 2. Materials and Methods

### 2.1. Sensor Placement and Measurement Setup

**Figure 1:**
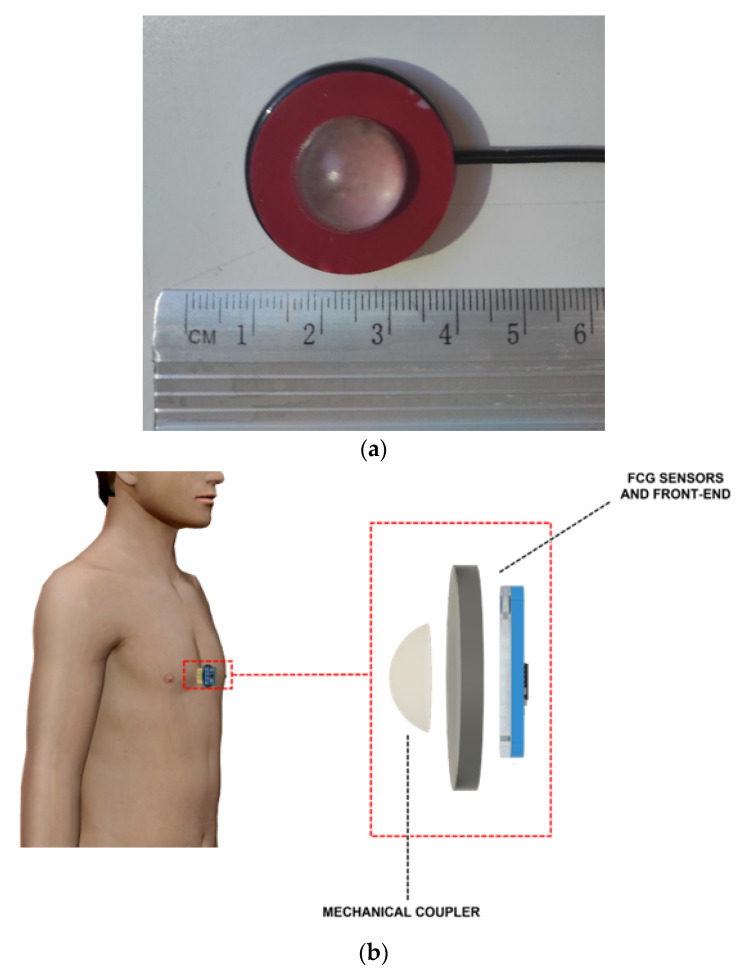
(**a**) Piezoelectric FCG sensor, (**b**) FCG sensor placement.

This article presents a retrospective analysis of the signals acquired during the study described in [22]. The materials and methodologies adopted for the experiments are reported below, but no measurements were carried out during this study. Figure 1a shows the piezoelectric sensor used in [22] to acquire the FCG signals, while Figure 1b shows sensor placement. The sensor was equipped with a dome-shaped mechanical coupler, as in [20,21,22,23], which ensures good mechanical transduction from the subject’s skin. The FCG sensor was placed on the chest of the subjects via medical adhesive tape, by roughly locating the point of maximal impulse (PMI), and then fastened with a belt around the thorax.

Six subjects (4 males and 2 females, age 36.6 ± 11.0 years) were comfortably seated on a chair, leaning against the seatback, while keeping their back straight. FCG acquisitions were performed via a National Instrument NI-USB4431 DAQ board (National Instruments Corp., 11,500 N Mopac Expwy, Austin, TX 78759-3504, USA), with 24-bit precision and 10 kHz sampling frequency. FCG recordings were acquired during quiet breathing.

### 2.2. Signal Processing

Raw FCG sensor signals are composed by a large component related to the breathing activity (the FRG), and a much smaller, superimposed component related to the cardiac activity (the actual FCG) [20,21,22,26,27]. Such components must be analyzed separately, so an efficient separation strategy is required. To this aim, the FRG component was first extracted, and then subtracted from the raw signal to isolate the actual FCG signal. As in [20,21,22,26,27], the FRG was extracted via a 3rd order Savitzki–Golay filter [28], with a frame length corresponding to an approximate interval of 1.5 s. As opposed common filters, the Savitzki–Golay filter fits signal portions of a given length with polynomials of a given order. This makes it well suited to separate a large signal from a much smaller oscillating signal, which is the case for the superimposed large respiratory component and the much smaller cardiac components of the raw FCG sensor signal. The actual FCG signal obtained by subtracting the FRG consists of two infrasonic components, namely LF-FCG and HF-FCG, and an audible component, corresponding to the heart sounds. The analyses presented in this study focused on the two infrasonic components, which were extracted from the FCG signal via band-pass filtering. In particular, 4th order zero-lag Butterworth band-pass filters with 0.6–5 Hz and 7–30 Hz frequency bands were used to extract the LF-FCG and HF-FCG components, respectively. In order to obtain information on the amplitude modulation of the LF-FCG and HF-FCG signals, the linear envelopes were extracted by performing rectification (absolute value), followed by a low-pass filtering via a 4th order zero-lag Butterworth filter with a cut-off frequency of 0.5 Hz. The linear envelopes were further filtered via the same 3rd order Savitzki—Golay filter used to extract the FRG signal.

Finally, the positive peaks related to inspiratory acts were located on both the FRG signals and the linear envelopes of the LF-FCG and HF-FCG signals to estimate the inter-breath intervals. Furthermore, respiratory acts in the FRG signal corrupted by motion artifacts and the corresponding acts in the linear envelopes of LF-FCG and HF-FCG signals were excluded from the analysis. All processing operations were performed in MATLAB^®^ R2018b (MathWorks, Inc., 1 Apple Hill Drive, Natick, MA, USA).

### 2.3. Statistical Analyses

Respiratory act detection in the linear envelopes of LF-FCG and HF-FCG signals was evaluated by computing the sensitivity and positive predictive value (PPV) as follows:

(1) $$\mathrm{Sensitivity}=\frac{\mathrm{TP}}{\mathrm{TP}+\mathrm{FN}}\;\cdot \;100$$

(2) $$\mathrm{PPV}=\frac{\mathrm{TP}}{\mathrm{TP}+\mathrm{FP}}\;\cdot \;100$$

where TP is the number of true positives, FN is the number of false negatives and FP is the number of false positives.

Moreover, to assess the consistency of the amplitude modulation of LF-FCG and HF-FCG signals within the respiratory cycle, regression, correlation, and Bland–Altman analyses were carried out on the inter-breath intervals obtained from FRG and the linear envelopes of LF-FCG and HF-FCG, by means of the MATLAB^®^ function “bland-altman-and-correlation-plot” [29].

## 3. Results

### 3.1. Morphological Comparison

**Figure 2:**
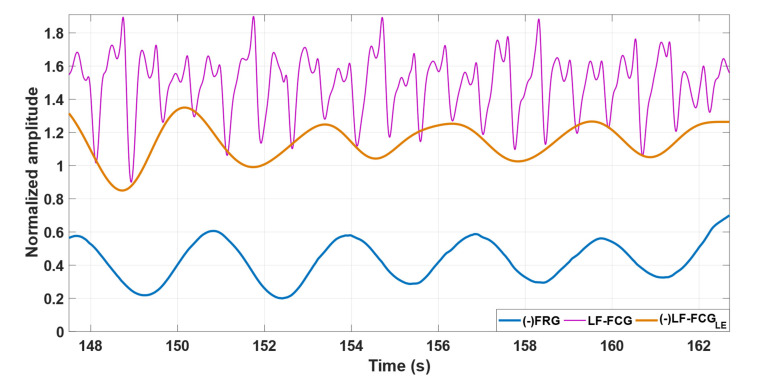
Some excerpts of FRG signal (blue line), LF-FCG signal (violet line) and its linear envelope (orange line) from subject #4.

Figure 2 depicts some excerpts of the FRG signal, LF-FCG signal and its linear envelope from subject #4. It can be noted by visual inspection that the amplitude of the LF-FCG signal (violet line) appears to be modulated by the respiration; indeed, its linear envelope (orange line) reflects the morphology of the FRG signal (blue line) very well.

**Figure 3:**
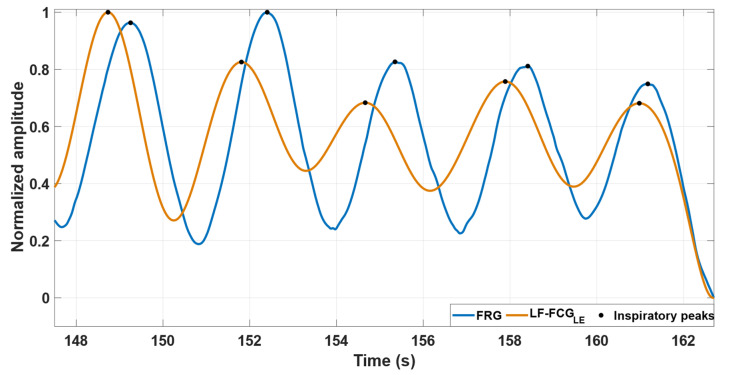
An excerpt of FRG signal (blue line) and the linear envelope of LF-FCG signal (orange line) from subject #4.

The good similarity between the FRG signals and the linear envelopes of LF-FCG signals is highlighted by Figure 3, which shows some excerpts of these signals from subject #4.

**Figure 4:**
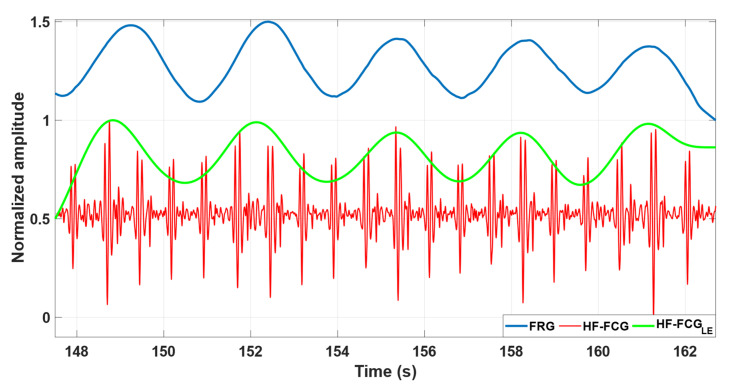
Some excerpts of FRG signal (blue line), HF-FCG signal (red line) and its linear envelope (green line) from subject #4.

Figure 4 depicts some excerpts of the FRG signal, HF-FCG signal and its linear envelope from subject #4. It can also be observed that the amplitude of the HF-FCG signal (red line) appears to be modulated by the respiration and its linear envelope (green line) reflects the morphology of the FRG signal (blue line) very well.

**Figure 5:**
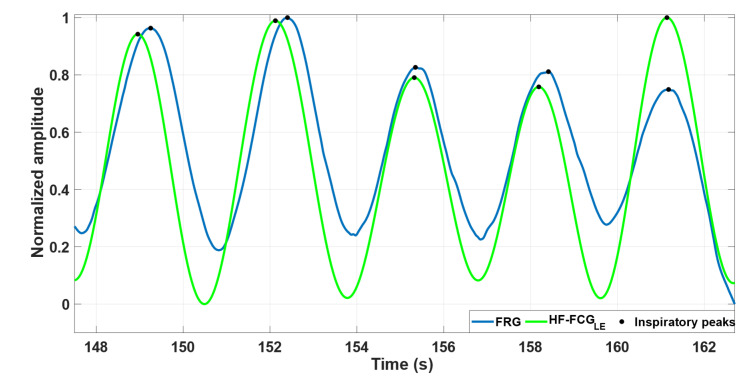
An excerpt of FRG signal (blue line) and the linear envelope of HF-FCG signal (green line) from subject #4.

The high similarity between the FRG signals and the linear envelopes of HF-FCG signals is highlighted by Figure 5, which shows some excerpts of these signals from subject #4.

**Figure 6:**
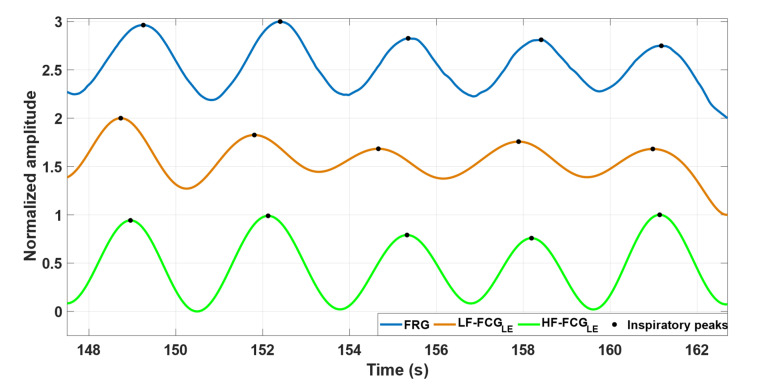
Some excerpts of FRG signal (blue line), the linear envelope of LF-FCG signal (orange line) and the linear envelope of HF-FCG signal (green line) from subject #4.

Figure 6 depicts the same excerpts of the FRG signal (blue line), the linear envelope of the LF-FCG signal (orange line) and the linear envelope of the HF-FCG signal (green line) from subject #4.

### 3.2. Statistical Analyses

Table 1 outlines the number of respiratory acts detected per subject in the FRG signal and in the linear envelopes of the LF-FCG and HF-FCG signals. The table also reports the number of missed and spurious respiratory acts identified in the linear envelopes of the LF-FCG and HF-FCG signals by assuming the FRG signal as the reference. A total of 272 respiratory acts were detected in the FRG signals, while 263 respiratory acts, 9 missed acts and 21 spurious acts were found in the linear envelope of LF-FCG signals, and 270 respiratory acts, 2 missed acts and 1 spurious act were found in the linear envelope of HF-FCG signals. Respiratory acts detected in the linear envelopes of both LF-FCG and HF-FCG signals were considered as true positives (TP), while missed and spurious acts were considered as false negatives (FN) and false positives (FP), respectively. Hence, the LF-FCG scored a sensitivity of 96.7% and a positive predictive value (PPV) of 92.6%, while the HF-FCG scored a sensitivity of 99.3% and a PPV of 99.6%. Sensitivity and PPV of respiratory act detection in the linear envelopes of LF-FCG and HF-FCG signals are also reported in Table 2.

**Figure 7:**
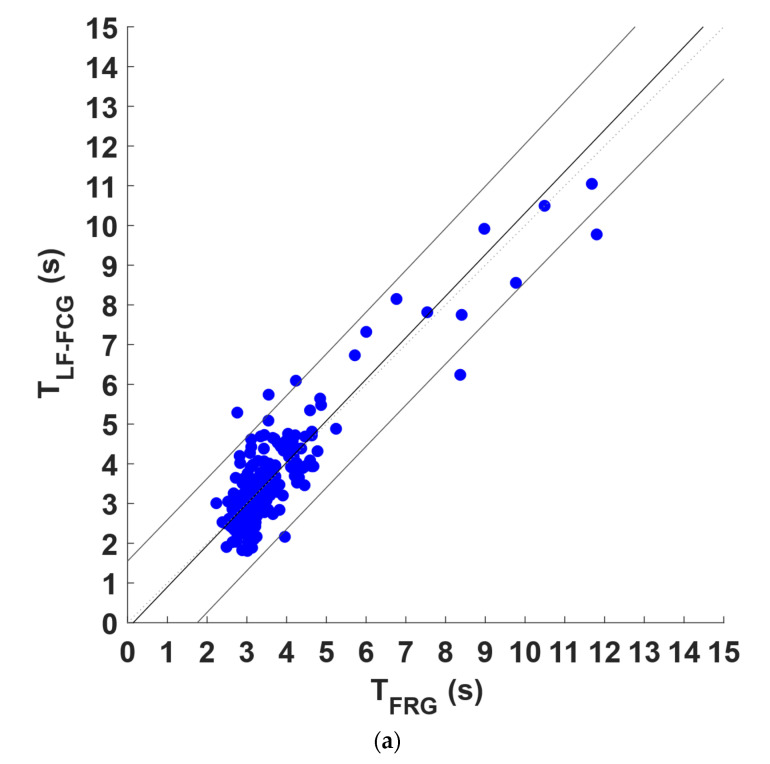

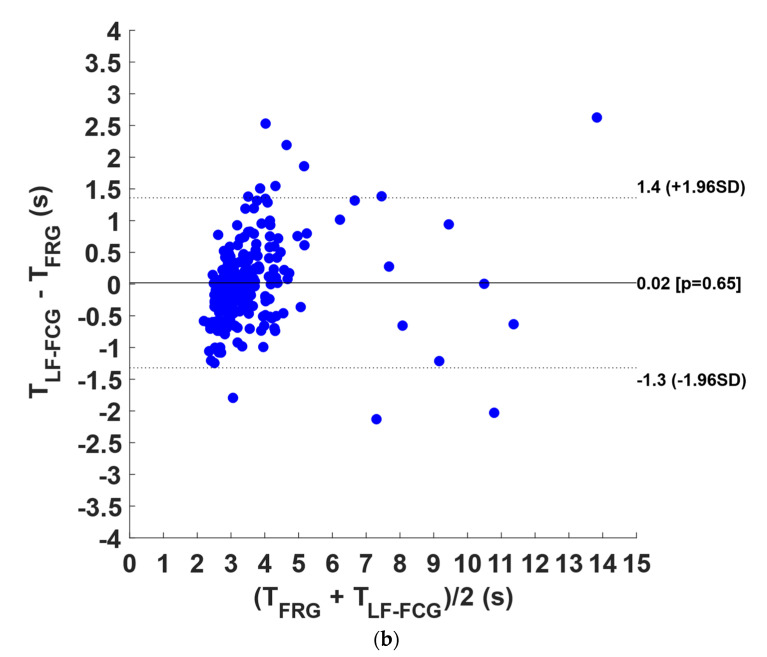
Statistical analyses of inter-breath intervals: (**a**) results of regression and correlation analyses; (**b**) results of Bland–Altman analysis for LF-FCG signals.

**Figure 8:**
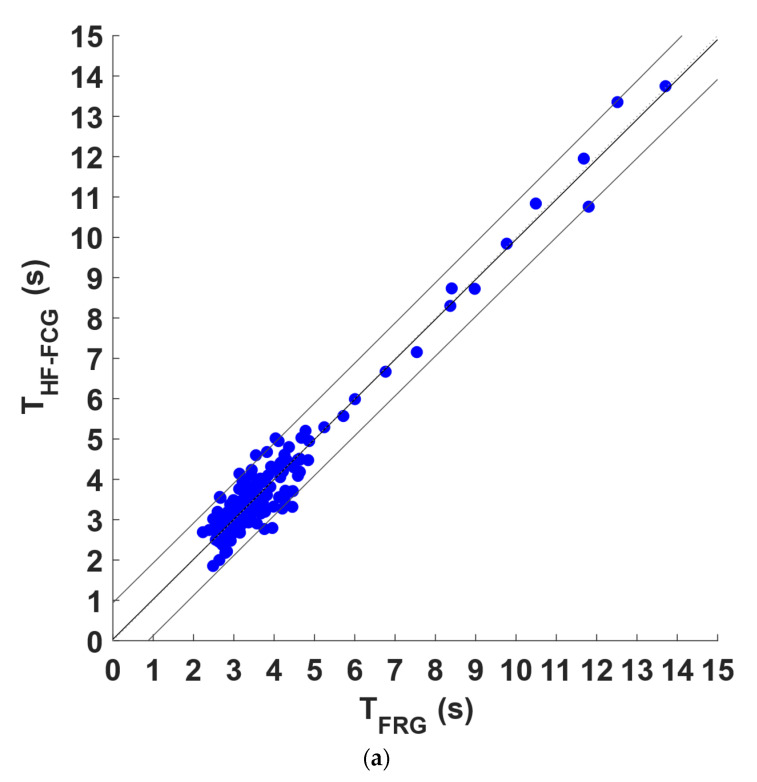

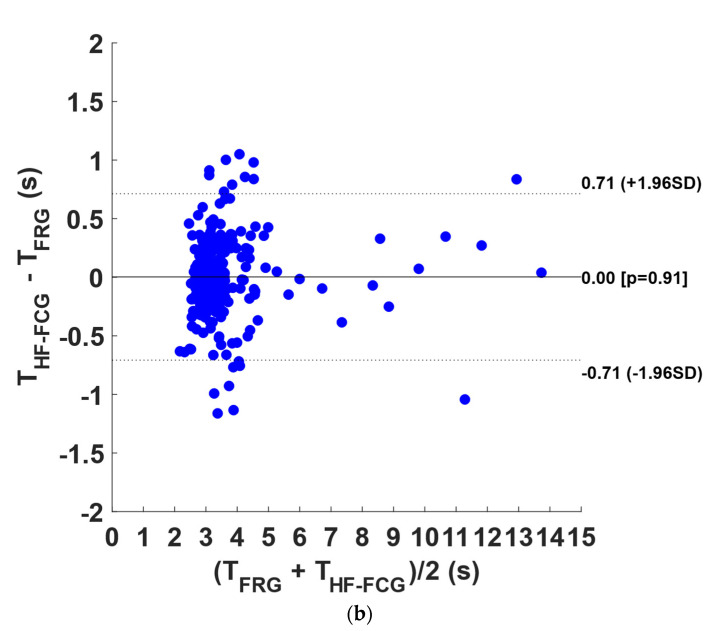
Statistical analyses of inter-breath intervals: (**a**) results of regression and correlation analyses; (**b**) results of Bland–Altman analysis for HF-FCG signals.

Regression, correlation, and Bland–Altman analyses were carried out on the inter-breath intervals related to the respiratory acts detected in the FRG signals and in the linear envelopes of the LF-FCG and HF-FCG signals. To this end, the intervals related to the missed and spurious respiratory acts in the linear envelopes of the LF-FCG and HF-FCG and the corresponding intervals in the FRG signals were excluded from the analysis. The statistical analyses were performed on 241 inter-breath intervals and the results are depicted in Figure 7 for LF-FCG and Figure 8 for HF-FCG. The regression and correlation analyses reported a slope and intercept of 1.05 and −0.147 s, with an R^2^ value of 0.86 for LF-FCG, while a slope and intercept of 0.991 and 0.0350 s, with an R^2^ value of 0.95 for HF-FCG. The Bland–Altman analysis reported a non-significant bias (*p* = 0.65) with limits of agreement (LoA) of ±1.34 s for LF-FCG, while a non-significant bias (*p* = 0.91) with LoA of ±0.710 s for HF-FCG. The results of regression, correlation and Bland–Altman analyses are also summarized in Table 3.

The statistical analyses were also performed on LF-FCG signals obtained by extending the filtering bandwidth from 0.6–5 Hz to 0.6–7 Hz. However, no substantial differences were observed in the obtained results.

## 4. Discussion

This preliminary study investigated the effect of respiration on LF-FCG and HF-FCG signals. To this end, FCG recordings were acquired on six healthy subjects at rest, during quiet breathing. First, FRG, LF-FCG and HF-FCG components were extracted from the raw FCG sensor signals. Then, the linear envelopes of LF-FCG and HF-FCG signals were computed to quantify their amplitude modulation. For all the subjects considered in this study, the heart rate was always more than two times the respiratory rate. This ensured the correct extraction of amplitude modulation, since the Nyquist–Shannon criterion was always met.

As shown in Figure 2 and Figure 4, the amplitude of the LF-FCG and HF-FCG signals appeared to be modulated by respiration. Various studies on SCG [8,9,30,31,32] and heart sounds [33,34,35,36] have found consistent variation in the amplitude of signals during various phases of the respiratory cycle, and nearly no variability during apnea. These results are consistent with those obtained from this study. During respiration, the position of the heart varies considerably within the chest, while the sensors placed on the surface maintain their position. Therefore, the mutual distance between the heart and the sensors varies in accordance with the respiratory phases, so the mechanical waves produced by the heart travel along paths of different lengths. Moreover, these cardiac mechanical waves have to pass through portions of tissues with different orientation and mechanical properties. These considerations may account for the variation in the amplitude of the received signals and also for small variations in the signal morphology.

To quantitatively analyze the relationship between the amplitude modulation of FCG signals and the breathing activity, first, the respiratory acts were detected in the FRG signals and in the linear envelopes of the LF-FCG and HF-FCG signals, and then the related inter-breath intervals were computed and compared via statistical analyses. The respiratory acts were recognized with sensitivity of 96.7% and PPV of 92.6% in the linear envelope of LF-FCG, and with sensitivity of 99.3% and PPV of 99.6% in the linear envelope of HF-FCG. As shown in Figure 7 and Figure 8, the statistical analyses reported a slope and intercept of 1.05 and −0.147 s (R^2^ = 0.86), as well as non-significant bias (*p* = 0.65) with LoA of ±1.34 s for LF-FCG, while a slope and intercept of 0.991 and 0.0350 s (R^2^ = 0.95), as well as non-significant bias (*p* = 0.91) with LoA of ±0.710 s for HF-FCG. These preliminary results suggest that the amplitude modulation of both LF-FCG and HF-FCG signals has good consistency within the respiratory cycle. It is worth noticing that the amplitude modulation of LF-FCG exhibited a lower consistency than that of HF-FCG, because the LF-FCG scored a lower R^2^ value and almost doubled limits of agreements. Respiratory-induced amplitude modulation of HF-FCG was expected, since it has already been observed in SCG signals [30].

### Limitations

This proof-of-concept study has some limitations. Only a few subjects were involved in the investigation because it was based on a retrospective analysis of the data acquired during a previous study [22]. Therefore, the preliminary results obtained need to be assessed on a larger cohort of subjects. In addition, the analyzed data had been acquired from subjects at rest, comfortably seated, performing quiet respiration. The amplitude modulation of the FCG signal components should also be assessed in different experimental conditions, e.g., by using different FCG sensor numbers and placement sites, changing subjects’ postures, during forced respiration and physical activities.

## 5. Conclusions

The preliminary results of this study show that the respiratory activity causes amplitude modulations of the infrasonic components of FCG signals. Indeed, a simple processing method provided amplitude modulation signals that closely matched the reference respiratory signal, as shown by the accurate localization of respiratory peaks and estimation of the related inter-breath intervals. These findings are in line with the results of previous studies on SCG and heart sounds, thus providing further support to the hypothesis that the amplitudes of cardiac vibrations are modulated during the respiratory cycle. Indeed, the heart, which is attached via the pericardium to the diaphragm, moves up and down during the respiratory cycle, so the distance between the heart and the surface mechanical sensors varies in accordance with the respiratory phase. As a result, the mechanical waves produced by the heart propagate through portions of tissues with different orientation and mechanical properties, along paths of variable length, thus being subject to variable amplitude attenuation.

Future studies could focus on amplitude modulation and possible morphological changes in other FCG components (e.g., HS-FCG), also investigating potential differences between FCG signals recorded from different sites on the chest. These analyses could also be extended to different experimental conditions, such as subjects in standing or supine positions, while performing voluntary tachypnea or bradypnea, during or after physical exertion. Understanding the relationship between breathing activity and the morphological changes in FCG signals in healthy subjects could pave the way for the identification of potential alterations in this relationship in pathological subjects, which could eventually be used for diagnostic purposes.

## Figures and Tables

**Table 1:** Respiratory acts detected in the FRG signal and the linear envelopes of LF-FCG and HF-FCG signals per subject. The missed and spurious acts are reported for the linear envelopes of LF-FCG and HF-FCG signals with reference to the acts detected in the FRG signal.

	Respiratory Acts			Missed Acts		Spurious Acts	
Subject	FRG	LF-FCG	HF-FCG	LF-FCG	HF-FCG	LF-FCG	HF-FCG
#1	57	58	57	4	0	5	0
#2	21	28	21	3	0	10	0
#3	83	86	83	0	1	3	1
#4	52	53	52	2	0	3	0
#5	18	18	18	0	0	0	0
#6	41	41	40	0	1	0	0
**Total**	272	284	271	9	2	21	1

**Table 2:** Sensitivity and PPV of respiratory act detection in the linear envelopes of LF-FCG and HF-FCG signals.

	Sensitivity (%)	PPV (%)
LF-FCG	96.7	92.6
HF-FCG	99.3	99.6

**Table 3:** Results of regression, correlation and Bland–Altman analyses for LF-FCG and HF-FCG signals.

	Slope	Intercept (s)	R^2^	Bias	*p*-Value	LoA (s)
LF-FCG	1.05	−0.147	0.86	Non-significant	0.65	±1.34
HF-FCG	0.991	0.0350	0.95	Non-significant	0.91	±0.710

## Data Availability

The data presented in this study are available upon request from the corresponding author.
